# Mucin glycoarray in gastric and gallbladder epithelia

**DOI:** 10.1186/1477-3163-6-10

**Published:** 2007-06-12

**Authors:** Iniya Meenakshi Ganesh, Duraibabu Subramani, Devaraj Halagowder

**Affiliations:** 1 Unit of Biochemistry, Department of Zoology, University of Madras, Life Sciences Building, Guindy Campus, Chennai – 600025, India

## Abstract

**Background:**

Mucins are critical cytoprotective glycoproteins and alterations of epithelial gastric mucins have been described in different pathological conditions. The purpose of the present study was to evaluate the putative usefulness of mucins in understanding the progression of gastric cancer and gallstone formation in a better perspective.

**Methods:**

Formalin-fixed paraffin-embedded gastric biopsy specimens and surgically resected gallbladder tissue samples were sectioned. Alcian Blue (AB) staining was performed to identify sialomucins (staining blue at pH 2.5) and sulfomucins (staining brown at pH 1.0) and then Periodic acid-Schiff's (PAS) staining to visualize the neutral mucins (staining magenta).

**Results:**

In normal gastric and gallbladder mucosae, we found that neutral mucins were predominant, whereas in intestinal metaplasia, gastric carcinoma and stone-containing gallbladder, a significant increase of acidic mucins was found.

**Conclusion:**

We suggest that the sulfomucins have a greater role in gallstone formation than the neutral mucins and also that the sialomucins and sulfomucins play an important role in cancer progression and metastasis. Our results challenge the glycobiologists to delve deeper in elucidating the role of mucins in gastric malignancy and in gallstone formation.

## Background

Mucins are expressed by various epithelial cell types that exist in relatively harsh environments [1]. Changes in the expression levels and glycosylation of mucins have been associated with several diseases, including carcinomas [2,3]. In gastric cancer, alterations in mucin expression have been reported: increased mucin heterogeneity [4] and glycosylation changes including exposure of simple mucin-type carbohydrates [5]. Mucin histochemistry has been used to characterize these transformations of normal gastric epithelium leading to intestinal metaplasia and to carcinoma [6]. These observations suggest that the repertoire of mucins synthesized by gastric carcinoma cells is tightly associated with their differentiation. The pattern of mucin expression may therefore provide new insights on the differentiation pathways of gastric carcinoma.

The gallbladder mucus plays a regulatory role in cholelithiasis as it promotes the nucleation of stones [7]. Mucus, calcium and lipids act in concert to form the cholesterol gallstones [8]. However, there is not on record a systematic study on the putative relationship between mucin carbohydrate changes in gastric cancer and black pigment gallstone formation. Since histochemical methods offer an excellent research tool for the characterization of glycoproteins [9] we attempted to investigate the alterations in these oligosaccharidic side chains in gastric and gallbladder epithelial cells by histochemical techniques to shed further light in elucidating the development of gastric carcinoma and gallstones.

## Methods

### Samples

Forty-four endoscopic human gastric biopsies of which five samples were normal, thirteen intestinal metaplasia and twenty-six carcinoma, and thirty surgically resected human gallbladders of which three were normal and twenty-seven were stone- containing, were obtained from individuals of Surgical Gastroenterology Unit, Stanley Govt. Medical College Hospital, Chennai after obtaining the ethical clearance of the Hospital Medical Board. All the specimens were fixed in 10% buffered formalin and routinely embedded in paraffin wax. Serial sections of 4 μm thickness were cut and used for histochemistry.

### Mucin Histochemistry

Alcian Blue (AB) staining was performed followed by Periodic acid-Schiff's (PAS) to distinguish between neutral mucins (staining magenta by PAS) and sialomucins (staining blue by AB at pH 2.5) and sulfomucins (staining brown by AB at pH 1.0).

The slides were dewaxed in xylene and treated in descending grades of ethanol (100%, 90%, 70%, 50% and 30%). For the PAS-AB technique, slides were rinsed in 3% acetic acid for 1 minute and treated with Alcian blue G8X(pH 2.5) for one hour and the procedure was repeated with Alcian blue G8X (pH 1.0) for sulfomucins. After washing in running tap water, the slides were treated with 1% periodic acid for 30 minutes and kept in dark. They were washed again in running tap water, treated with Schiff's reagent for one hour and kept in dark conditions [6]. The slides were finally dehydrated in ascending series of ethanol (30%, 50%, 70%, 90% and 100%) cleared in xylene and mounted. After drying, the sections were visualized in Axioscope two plus microscope (Carl Zeiss).

## Results

### Mucin histochemistry of normal gastric epithelium

**Figure 1:**
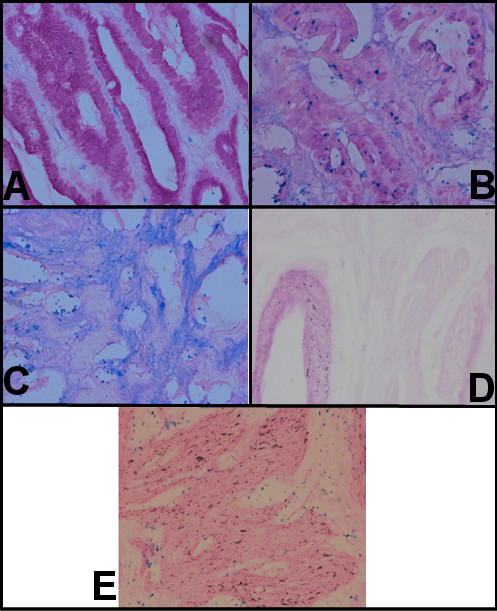
**(A) **Histochemical analysis of neutral mucins, sialomucins and sulfomucins in gastric and gallbaldder epithelia. Normal Gastric Epithelium (× 40).**(B) **Histochemical analysis of neutral mucins, sialomucins and sulfomucins in gastric and gallbaldder epithelia.**(C) **Histochemical analysis of neutral mucins, sialomucins and sulfomucins in gastric and gallbaldder epithelia. Gastric Carcinoma (× 40).**(D) **Histochemical analysis of neutral mucins, sialomucins and sulfomucins in gastric and gallbaldder epithelia. Normal gallbladder epithelium (× 40).**(E) **Histochemical analysis of neutral mucins, sialomucins and sulfomucins in gastric and gallbaldder epithelia. Stone-containing gallbladder epithelium (× 40).

Neutral mucins were expressed in the foveolar epithelium and in the mucus gland cells of the antrum. Sialomucins were slightly detected in normal gastric mucosa. An occasional staining of sulfomucins was found in the foveolar epithelial cells as shown in fig.1A.

### Mucin histochemistry in intestinal metaplasia

Neutral mucins were decreased in intestinal metaplasia compared to normal gastric mucosa, whereas sulfomucins were found to be very high and intense staining of alcian blue shows increased expression of sialomucins as in fig. 1B.

### Mucin histochemistry in gastric carcinoma

Fig. 1C shows a significant staining of sialomucins rather than sulfomucins by alcian blue staining performed at pH 2.5 and pH 1.0 respectively in gastric carcinoma compared to normal gastric mucosa and a mild staining of neutral mucins by PAS.

### Mucin histochemistry of normal gallbladder

With the PAS-AB method, most of the goblet cells of normal gallbladder epithelium stained magenta indicating the presence of neutral mucins and the acidic mucins showed mild blue staining by alcian blue but brown color staining were not observed indicating the absence of sulfomucins as in fig. 1D.

### Mucin histochemistry of stone-containing gallbladder epithelium

Sulfomucins were expressed in supranuclear portions with strong intensity as shown by the brown staining of alcian blue at pH 1.0 and the intense blue staining of sialomucins along with the weak staining intensity of neutral mucins in goblet cells was observed by PAS-AB at pH 2.5 which is shown in fig 1E.

## Discussion

Mucins are expressed in a cell- and tissue-specific pattern in normal tissue [2]. Alterations of the expression pattern of mucins have been described in carcinomas as well as in their precursor lesions [10] and in the formation of gallstones [11]. In intestinal metaplasia, altered mucin carbohydrate and peptide moieties of mucins may constitute molecular markers of an increased risk of malignant transformation [12].

In agreement with previous studies reporting the distributions of mucins in normal stomach and gallbladder [13-15], we found that the expression of neutral mucins was predominant. Intestinal metaplasia is one of the lesions identified in the cascade of events that precede the development of gastric cancer [16]. By histology, the intestinal metaplasia is identified by the substitution of gastric mucosa by an epithelium that resembles the intestinal mucosa. We have observed moreover that sialomucins are highly expressed in intestinal metaplasia, gastric carcinoma as reported by Filipe *et al*., [17] and stone-containing gallbladder epithelium [11].

Altered hepatic bile supersaturated with cholesterol, nucleation of cholesterol monohydrate crystals and impaired gallbladder functions are three major factors determining the formation of cholesterol gallstones [18]. Nucleation of cholesterol monohydrate crystals is closely related to the concentration of mucus glycoproteins [19]. However, in black pigment stones which are devoid of cholesterol, the interaction of mucin, calcium and bilirubin is the sole mechanism for cholelithiasis [20]. In this context the role of acidic mucins, especially sulfomucins becomes more important to the formation of black pigment stones. Since the presence of sulfomucins in black pigment stone-containing gallbladder epithelium has not yet been clearly established, we have analyzed the same and report that sulfomucins are significantly expressed in stone-containing gallbladder epithelium compared to normal gallbladder epithelium and even to intestinal metaplasia. This may indicate that patients with gallstone disease are more prone to gallbladder cancer than those without stones.

Therefore, our results may contribute to clarify the changes from neutral mucins to acidic mucins in gastric and gallbladder epithelium. The expression of sialylated and sulfated residues is not dependent upon the protein core of mucin. It remains to be investigated whether all mucins (MUC1, MUC2, MUC4, MUC 5AC, MUC 5B, MUC6 etc.) [21,22] may carry these sialylated and sulfated oligosaccharidic sidechains detected in intestinal metaplasia, gastric carcinoma and cholelithiasis.

## Conclusion

We conclude that the sulfomucins have a greater role in gallstone formation than the neutral mucins showing that patients suffering from gallstone disease have a high risk for gallbladder cancer and also that the sialomucins and sulfomucins play an important role in cancer progression and metastasis. Our results challenge the glycobiologists to delve deeper in elucidating the role of mucins in gastric malignancy and in gallstone formation. Drugs or compounds that regulate the sialylation and sulfation might be an effective way to inhibit invasion, metastasis and gallstone formation and this may open up novel therapeutic approaches.

## List of abbreviations

PAS: – Periodic acid-Schiff

AB: – Alcian Blue.

## Competing interests

The author(s) declare that they have no competing interests.

## Authors' contributions

IMG has designed and carried out all the experiments and was also involved in drafting the manuscript in coordination with DS and DH. DS was involved in revising the manuscript critically for important intellectual content. DH has reviewed the histochemical results, provided the suggestions and has made corrections for the finalization of this paper. All the authors have read and approved the final manuscript.

## References

[B1] Forstner JF (1978). Intestinal mucins in health and disease. Digestion.

[B2] Ho SB, Niehans GA, Lyftogt C, Yan PS, Cherwitz DL, Gum ET, Dahiya R, Kim YS (1993). Heterogeneity of mucin gene expression in normal and neoplastic tissues. Cancer Res.

[B3] Corfield A, Myerscough N, Gough M, Brockhausen I, Schauer R, Paraskeva C (1995). Glycosylation patterns of mucins in colonic disease. Biochem Soc Trans.

[B4] Ho SB, Shekels LL, Toribara NW, Kim YS, Lyftogt C, Cherwitz DL, Niehans GA (1995). Mucin gene expression in normal, pre-neoplastic and neoplastic human gastric epithelium. Cancer Res.

[B5] Carneiro F, Amado M, David L, Clausen H, Sobrinho-Simões M (1994). Glycosylation features of gastric carcinoma initiation and progression. A review with emphasis on simple mucin-type carbohydrates and histo-blood group antigens of the Lewis. Eur J Cancer Prev.

[B6] Reis AC, David L, Correa P, Carnerio F, de Bolós C, Garcia E, Mandel U, Clausen H, Sobrinho-Simões M (1989). Intestinal metaplasia of HumanStomach displays distinct patterns of mucin (MUC1, MUC2, MUC 5AC, MUC 6) expression. Cancer Res.

[B7] Afdhal NH (1990). Choleterol crystal nucleation: A decade- long search for the missing link in gallbladder pathogenesis. Hepatology.

[B8] Jacyna MR (1990). Interactions between gallbladder bile and mucosa; Relevance to gallstone formation. Gut.

[B9] Madrid JF, Hernández F, Ballesta J (1997). Characterization of glycoproteins in the epithelial cells of human and other mammalian gallbladder: A review. Microsc Res Tech.

[B10] Hakomori S (1989). Aberrant glycosylation in tumors and tumor-associated carbohydrate antigens. Adv Cancer Res.

[B11] Lee KT, Liu TS (2001). Mucin gene expression in gallbladder epithelium with black pigment stone ascertained by *in situ *hybridization. The Kaohsiung J Med Sc.

[B12] Springer GF, Desai PR, Ghazideh M, Tegtmeyer H (1995). T/Tn pancarcinoma autoantigens: fundamental diagnosis and prognosis aspects. Cancer Detect Prev.

[B13] Filipe MI Jass JR, Filipe MI, Jass JR (1989). Intestinal Metaplasia subtypes and cancer risk. Gastric Carcinoma.

[B14] Filipe MI, Fenoglio-Preiser CM, Wolfe M, Rilke F (1992). Borderline lesions of the gastric epithelium: new indicators of gastric risk and clinical implications. Progress in Surgical Pathology.

[B15] Sheen PC, Lee KT, Liu YE (1998). Mucin content in gallbladders with brown pigment stones or combination stones with a brown stones with a brown periphery. Digestion.

[B16] Correa P (1980). A human model of gastric carcinogenesis. Cancer Res.

[B17] Filipe MI, Muños N, Matko I, Kato I, Pompe-Kirn V, Jutereseek A, Teuch-mann S, Benz M, Prijon T (1994). Intestinal Metaplasia types and risk of gastric cancer. Int J Cancer.

[B18] Sherlock S, Dooley J, Sherlock S, Dooley J (1992). Gallstones and inflammatory gallbladder disease. Diseases of the Liver and Biliary System.

[B19] Groen AK, Noorddam C, Drapers JAG, Egbers P, Jansen P, Tytgat GNT (1990). Isolation of a potent cholesterol nucleation promoting-activity from human gallbladder bile: Role in the pathogenesis of gallstone disease. Hepatology.

[B20] Afdhal NH, Ostrow JD, Rege RV, Dawes LG (1990). Interaction of bovine gallbladder mucin and calcium-binding protein effects on calcium phosphate precipitation. Gastroenterology.

[B21] de Bolós C, Real FL, Ferrer AL (2001). Regulation of mucin glycoconjugate expression from normal epithelium. Frontiers in Bioscience.

[B22] Duraibabu S, Jayanthi V, Niranjali S, Celso AR, Devaraj H (2006). Expression profile of mucins (MUC 2, MUC 5AC and MUC 6) in Helicobacter pylori infected preneoplastic and neoplastic gastric epithelium. Molecular Cancer.

